# Preservation of freshly-cut lemon slices using alginate-based coating functionalized with antioxidant enzymatically hydrolyzed rice straw-hemicellulose

**DOI:** 10.1038/s41598-024-77670-6

**Published:** 2024-11-08

**Authors:** Shaymaa A. Ismail, Safaa S. Abozed, Hanan A. A. Taie, Amira A. Hassan

**Affiliations:** 1 Department of Chemistry of Natural and Microbial Products, Pharmaceutical and Drug Industries Research Institute, National Research Centre, 33 El Bohouth St., Dokki, 12622 Giza, Egypt; 2 Food Technology Department, Food Industry and Nutrition Research Institute, National Research Centre, 33 El Bohouth St., Dokki, 12622 Giza, Egypt; 3 Plant Biochemistry Department, Agricultural and Biology Research Institute, National Research Centre, 33 El Bohouth St., Dokki, 12622 Giza, Egypt

**Keywords:** Antioxidant activity, Bioactive compound, Bioresources, Edible coatings, Lemon slices shelf-life, Biotechnology, Plant sciences

## Abstract

Food coatings are efficient preservative measures, a crucially needed approach to meet hunger growth as well as food management. In the current study, the construction of an efficient coating using alginate polymer fortified with antioxidant rice straw-hemicellulose hydrolysate was examined. Rice straw hemicellulose fraction was extracted under thermal alkaline conditions with a recovery percentage of 15.8%. The extracted hemicellulose fraction was enzymatically hydrolyzed with microbial xylanase with hydrolysis percentage of 53.8%. Characterization of the produced hydrolysate was performed with the aid of thin layer chromatographic analysis (TLC), high-performance liquid chromatographic analysis (HPLC), X-ray diffraction (XRD), and Fourier transform infrared (FTIR) analysis. The reported data showed that xylobiose (240.68 mg/g) in addition to coumaric (383.33 µg/g) and ferulic acid (298.77 µg/g) as the main constituents of the carbohydrate and the polyphenolic contents, respectively. The hydrolysate possessed antioxidant capacity that significantly increased in a direct correlation with the concentration of the hydrolysate. Finally, the prepared coating solution effectiveness in the preservation of lemon slices against fungal growth was monitored up to 20 days with a significant concentration dependent decrease in weight loss and an increase in its antioxidant activity. The combination of xylooligosaccharide-rich rice straw hydrolysate with alginate-based coating not only improved the storage shelf-life of fresh fruits and vegetables but also provided microbial safety and potential benefits for human health.

**Supplementary Information:**

The online version contains supplementary material available at 10.1038/s41598-024-77670-6.

## Introduction

Food loss represents a serious crisis that exacerbates the global hunger management and according to a report by the United Nations Food and Agriculture Organization, more than one-third of the world’s food produced for human consumption is deteriorated annually^1^. Therefore, there is a significant demand to progress a viable food preservation strategy. Edible film application and coating have been presented as effective and promising methods for food preservation that minimize microbial growth and extend the shelf life of the food product^2^. These films and coatings are biopolymer-based materials with a thickness less than 0.3 mm^3^. Edible films are initially prepared and then adhered to the final food product, while coating is based on immersion of the food product in the coating material. Both of them have been applied for the preservation of various food products including vegetables, fruits, cheese, fish, meat, and snacks^2,4^.

Generally, coatings materials and edible films have been categorized into 3 major categories based on the physicochemical properties of their constituents; hydrocolloid (polysaccharide and protein), lipid colloid, and composite in which most of the applied materials are of natural origin as they are biocompatible and biodegradable with minimal reported toxicity^5^. Several studies indicated the suitability of several natural polysaccharides including cellulose derivatives, starch, pectin, chitosan, gums, and seaweed polysaccharides as packaging materials providing enhanced positive impact on the environment as they are non-toxic and biodegradable^6^. Alginate is a seaweed polysaccharide constituted of linear chains of β-D-mannuronic and α-L-guluronic acids linked by (1,4)-bonds^7^. Application of alginate as food additives has been accepted by the Food and Drug Administration (FDA) as well as the European Commission. Moreover, the unique colloidal properties of alginates enable the formation of transparent and uniform films^8^. Incorporation of bio-active compounds within the alginate matrix has addressed added value to the prepared films referring to their antimicrobial, anti-browning and antioxidant efficiency which prolong the shelf-life of the food product^9,10^. Moreover, the phenolic compounds improve the synthetic films mechanical, barrier, and physico-chemical qualities^11^.

Recently, advanced research has been focused on the utilization of agrifood byproducts to construct edible films and coatings. These byproducts are regarded as valuable resources that offer a variety of polymers, such as proteins and polysaccharides, as well as bioactive substances with diverse properties including antioxidant, antimicrobial and others, that can enhance the performance of the manufactured film and confer health benefits to the consumers^12^. Rice is one of the greatest popular food harvests cultivated globally in which about 1.35 tons of rice straw byproduct were produced from every ton of harvested rice grain^13^. Rice straw byproduct is a lignocellulosic biomass composed of 38.3% cellulose, 31.6% hemicellulose, 11.8% lignin, and 18.3% ash^14^. It has been reported that only 20% of the global production of rice straw is utilized ecofriendly, leaving more than 100 million tons being burned annually with negative environmental and health impacts^15^.

Several studies estimated rice straw as a promising source for a variety of products that can be efficiently utilized in food production sector^16–19^. In general, hemicellulose is a branched hetero-polysaccharide in which xylose is the main constituent with the distribution of various sugars such as mannose, arabinose and galactose^20^. The hemicellulose content, its chemical structure, and consequently its bioactivity varies with the variation of the biomass origin. The preliminary step for the utilization of the hemicellulose is its effective fractionation from other constituents of the plant cell wall. Several techniques including solvent extraction, steam explosion and hot water extraction in addition to microwave, ultrasonic treatment and enzymatic hydrolysis have been recorded. Alkaline extraction was the most commonly applied method^21^. Furthermore, the extracted hemicellulose was hydrolyzed applying thermo-chemical techniques or enzymes, while the enzymatic hydrolysis is preferable in the food and pharmaceutical industries^22^. As a result of hemicellulose hydrolysis, various antioxidant oligosaccharides have been produced^23^. Integration of edible films with oligosaccharides has been reported as a value-added product concerning the technological and functional aspects of the used polymers^24^.

Lemon is one of the most important citrus species, rich in natural constituents including vitamin C, phenolic compounds, essential oils, dietary fiber, citric acid and carotenoids that give its nutritional, health, and economic fundamentality^25^. In order to reduce the spoilage, metabolism and deterioration of fresh fruits in addition to extend their shelf-life during storage, it was investigated how to create an effective coating using alginate polymer enhanced with antioxidant rice straw-hemicellulose hydrolysate. The extraction of rice straw hemicellulose was conducted followed by its enzymatic hydrolysis using *Aspergillus terreus* xylanase. Characterization of the produced hydrolysate concerning its reducing sugars and phenolic content was carried out. In addition, its cytotoxicity and antioxidant activity were evaluated. Moreover, its applicability for the preparation of alginate films and its influence on the physico-chemical characteristics of the prepared films was examined. Finally, the prepared hydrolysate-alginate coating solution was tested for the preservation of lemon slice samples.

##  Materials and methods

### Materials

3,5-Dinitrosalicylic acid (DNS), Folin–Ciocâlteu reagent, butylated hydroxytoluene (BHT), 2,2-diphenyl-1-picryl hydrazyl radical (DPPH), 2,2’-azino-bis (3-ethylbenzothiazoline-6-sulfonic acid (ABTS), gallic acid, rutin, Sodium alginate, silica gel 60 TLC were purchased from Sigma Aldrich (Merck, Darmstadt, Germany). Human normal fibroblast cell line (BJ1) was obtained from the American type culture collection, Rockville, MD, USA.

### Extraction of rice straw hemicellulose fraction

#### Extraction process

The collected rice straw obtained from fields situated in Al Sharqiya, Egypt, was grinded, cleaned with water and dried at 50 ºC for 24 h. The hemicellulose fraction was extracted based on Chen and Anderson method^26^ with minor modifications. The rice straw sample (100 g) was soaked for 24 h in 1 L of 4.0% NaOH solution. After that, it was filtered through cotton mesh, and its pH was adjusted by HCl concentrated solution to 5. The solubilized hemicellulose fraction was precipitated through the addition of ethanol to the hemicellulose fraction (ratio 2:1) and left at room temperature for 24 h. The resulted hemicellulose precipitate was filtered, washed several times with ethanol, and left to dry in a pre-weighted Petri dish. The recovery percentage was estimated as follow:

$${\text{Recovery}}\;{\text{percentage}}\left( \% \right) = ({\text{P}}_{{{\text{After}}}} {-}{\text{P}}_{{{\text{Before}}}} )/{\text{Total}}\;{\text{weight}}\;{\text{of}}\;{\text{rice}}\;{\text{straw}} \times {\text{1}}00$$

where; P_After_ was the weight of the Petri dish after drying the extracted hemicellulose fraction and P_Before_ was the weight of the free Petri dish.

#### Impact of thermal treatment

The effect of the thermal treatment on the extraction efficiency of rice straw hemicellulose was tested by heating the extraction solution at 80 ºC for 1 h^27^. Moreover, the microwave pre-treatment of rice straw was evaluated by suspending the agro-residue sample in acetate buffer solution (200 mM, pH 5) and subjected to microwave for 1 min, washed several times with distilled water and dried at 50 ºC^28^.

#### Fourier transforms infrared spectroscopic analysis

FTIR spectrophotometer (Vertex 80v, Bruker, Berlin, Germany) was used to assess the active function groups and chemical bonds of the extracted hemicellulose fraction compared with commercial xylan sample in the range of 4000 –400 cm^−1^.

#### Microstructural analysis

A high-resolution field emission SEM (Quanta 250, HRFEG, Czech) was used to examine the morphological changes in the remaining residue of the treated rice straw samples. The gold-coated treated samples were scanned at 20 kV accelerating voltage and 1500 magnification power.

### Enzymatic hydrolysis of the extracted hemicellulose

#### Enzyme production

Xylanase was produced under solid-state conditions of rice straw-based fermentation media using the fungus *Aspergillus terreus*. The optimized production media contained 3.75 g rice straw moistened (1:3 ratio) with a moistening solution composed of (g/L): (NH_4_)_2_SO_4_; 10, KH_2_PO_4_; 2, CaCl_2_; 0.3, and MgSO_4_.7H_2_O; 0.3, adjusted to pH 7). Two milliliters of the spore suspension (nine-day-old pre-cultured slants) were added to the autoclaved production media, and it was then incubated for eight days at 30 ^ο^C. The fermented media was extracted with 50 mL distilled water for 1 h at 30 ^o^C and shaking speed 50 rpm. The clear supernatant was precipitated with ethanol (70% concentration) and the partially purified enzyme fraction was used in the next experiment^28^.

The xylanase activity was estimated according to modified method of Bailey et al.^29^, by monitoring the hydrolysis of 1% xylan solution (50 mM acetate buffer pH 5) for 30 min at 50 ºC where the released reducing sugars were assayed using DNS according to Miller^30^, in which xylose was used as a standard (Supplementary Fig. 1). One xylanase unit was identified as the quantity of the enzyme that frees one µmol of the reducing sugars per minute. In addition, the protein content of the fermentation media was measured according to Lowry et al.^31^ applying bovine serum albumin as a standard.

#### Hydrolysis process

Hydrolysis of the prepared hemicellulose was performed by adding an equivalent volume of 10% enzyme solution (40.796 Umg^−1^ protein) to the hemicellulose sample solution (2%, w/v in 50 mM acetate buffer pH 5) and incubated for 4 h at 40 °C. Finally, the catalytic action of the added enzyme was stopped by boiling the reaction mixture for 10 min followed by centrifugation at 4000 rpm and 4 °C for 10 min^28^.

The liberated reducing sugars were assayed spectrophotometrically at 540 nm using DNS reagent where the hydrolysis percentage was calculated as follow:

$${\text{Hydrolysis}}\;{\text{percentage}} = {\text{R/T*100}}$$

where, R refereed the quantity of the released reducing sugars, and T is the original hemicellulose added to the reaction medium. Moreover, clear supernatant after hydrolysis was analyzed via TLC using a mobile phase composed of propanol: water: ammonia (70:20:10, v/v) and diphenyl amine-aniline as spraying reagent^32^.

#### Characterization of the dried hydrolysate

##### Reducing sugars and phenolic content

Reducing sugars present in the air-dried hydrolysate sample (RS) and the parent compound (the extracted hemicellulose) was quantified according to the DNS method^30^. In addition, the phenolic content was assayed by Folin–Ciocâlteu method^33^ using gallic acid standard curve shown in supplementary Fig. 2.

##### Water solubility

The water solubility of both RS and the parent compound was carried out following Cano-Chauca et al.^34^ method. One gram of the tested samples was soaked in 99 mL of distilled water and subjected to 24-hour incubation period at 25 °C and 150 rpm. After centrifuging the sample solution for 20 min at 5500 rpm, 25 mL of the resultant clear supernatant were dried at 105 °C in a pre-weighted petri dish.

##### High performance liquid chromatographic analysis

The sugar components that present in RS sample were quantified by HPLC analysis (Agilent Technology 1100 series, using a refractive index detector, Shim-pack SCR-101 N separating column and ultra-pure water as the mobile phase with a flow rate of 0.7 mL/min). In addition, the phenolic compounds were analyzed by HPLC applying an Agilent Technology 1260 series accompanied with a multi-wavelength detector monitored at 280 nm and Eclipse C18 separating column using a mobile phase containing water and trifluoracetic acid (0.05% solution in acetonitrile) with a flow rate at 0.9 mL/min.

##### X-ray diffraction analysis

This was carried out using a Panalytical X’Pert PRO diffractometer with Cu Kα source and 10 − 70° scanning range.

### In vitro cytotoxicity of the dried hydrolysate

The cytotoxicity of RS was tested on BJ1 cell line according to Mosmann^35^, using 3-(4,5-dimethylthiazol-2-yl)-2, 5-diphenyl tetrazolium bromide (MTT). The percentage of change in cell viability was estimated as following:

$${\text{Viability}}\;{\text{change \% = (RS}}\;{\text{reading / Negative}}\;{\text{control}}\;{\text{reading)}}^{{ - 1}} \times 100$$

### Antioxidant activity

The antioxidant capability of RS was evaluated at concentration 250, 500, 750, and 1000 µg/ml using different assays in compare to the positive control BHA.

#### DPPH-free radical scavenging assay

DPPH free radical scavenging activity was determined in accordance with Brand-Williams et al.^36^ in which 0.5 ml of each concentration of the sample, 1.0 ml of freshly made methanolic DPPH solution (20 µg/ml^−1^), was added, and stirred. The decolorizing process was recorded at 517 nm and the following formula was used to determine the scavenging capacity:

$${\text{DPPH}}\;{\text{scavenging}}\;{\text{activity}}\;\left( \% \right) = \left( {{\text{control}}\;{\text{absorbance}} - {\text{sample}}\;{\text{absorbance}}} \right)/{\text{control}}\;{\text{absorbance}}) \times 100$$

where the control absorbance was the absorbance of DPPH solution and the sample absorbance was the absorbance of DPPH sample extract solution.

#### Reducing power assay

The reduction power of the sample was evaluated by combing 0.5 ml of each concentration of the sample with phosphate buffer (2.5 ml, 0.2 M, pH 6.6) and 1% potassium ferricyanide (2.5 ml). For twenty minutes, the mixture was incubated at 50 °C. After adding aliquots of trichloroacetic acid (2.5 ml, 10%) to the mixture, the mixture was centrifuged for 10 min at 1000 rpm. A freshly made FeCl_3_ solution (0.5 ml, 0.1%), together with 2.5 ml of distilled water, were combined with the upper layer of solution. At 700 nm, the blue-green color’s intensity was measured^37^.

#### ABTS radical scavenging activity

The ABTS radical scavenging activity was evaluated using the cation decolorization assay, which was adapted from the description provided by Re et al.^38^. The stock solutions were ABTS solution (7 mM) and potassium persulfate solution (2.4 mM). The working solution was then made by mixing the two stock solutions equally. It was then allowed to react for 12 h at room temperature in the dark. The spectrophotometer was then used to achieve an absorbance of 0.706 ± 0.001 units at 734 nm after diluting the solution by mixing 1 ml of ABTS radical solution with 60 ml of methanol. A newly prepared ABTS radical solution was used in each test. The 0.5 ml of the dry hydrolysate sample concentrations was mixed with 2.5 ml of the ABTS reagent. The absorbance at 734 nm was measured after 7 min using a spectrophotometer. The dry hydrolysate sample ability to decolorize ABTS radical cations in assay and the percentage inhibition determined as ABTS radical scavenging activity.

$${\text{ABTS}}\;{\text{scavenging}}\;{\text{activity}}\;\left( \% \right) = \left( {{\text{control}}\;{\text{absorbance}} - {\text{sample}}\;{\text{absorbance}}} \right)/{\text{control}}\;{\text{absorbance}}) \times {\text{1}}00$$

where the control absorbance was the absorbance of ABTS radical cation methanol and the sample absorbance was the absorbance of ABTS radical cation sample extract.

#### Ferric reducing ability of plasma assay

With a few modifications, the assay was carried out in accordance with Benzie and Strain^39^. The stock solutions were 20 mM FeCl_3_.6H_2_O solution, 300 mM acetate buffer at pH 3.6, and 10 mM of TPTZ (2,4,6-tripyridyl-s-triazine) solution in 40 mM HCl. Before utilizing, the freshly made working solution was heated to 37 °C by combining 25 ml of acetate buffer, 2.5 ml of TPTZ solution, and 2.5 ml of FeCl_3_.6H_2_O solution. For thirty minutes in the dark, 500 µl of the sample and 2500 µl of the prepared solution were allowed to react. At 593 nm, measurements of the colored product (ferrous tripyridyltriazine complex) were subsequently made. The findings were represented in terms of µmol Trolox/100 g dry sample.

#### Metal chelating activity assay

This assay depended on the estimation of the ferrous ions chelated by the sample in which 50 µl FeCl_2_ (2 mM) was mixed with 0.5 ml of the sample. After adding 200 µl of ferrozine (5 mM), the mixture was allowed to stand at room temperature for 10 min. The solution’s absorbance was measured spectrophotometrically at 562 nm once equilibrium had been reached^40^. The percentage of each sample’s ferrozine-ferrous complex inhibition was conducted using the following formula:

$${\text{Inhibition}}\;{\text{percentage}} = \left[ {\left( {{\text{sample}}\;{\text{absorbance}} - {\text{control}}\;{\text{absorbance}}} \right)/{\text{control}}\;{\text{absorbance}}} \right] \times {\text{1}}00$$

### Synthesis and characterization of alginate/rice straw-hemicellulose hydrolysate films

#### Preparation of the dipping and coating solutions

Alginate (Alg) solution was prepared via dissolving 2.5 g of sodium alginate powder into 100 mL water at 70 °C with continuous mechanical stirring forming a uniform dispersion followed by the addition of 1.0 g glycerol to the cooled alginate solution as a plasticizer. Likewise, different RS quantities, 0% (Alg), 1% (Alg/RS 1), 2.5% (Alg/RS 2.5), and 5% w/w (Alg/RS 5), were added into the above mixture and re-stirred for 15 min.

#### Characterization of the prepared films

Initially, 25 mL of the prepared matrix were dispensed into glass plates (90 × 15 mm) and dried at 60 °C in a vacuum drying oven for 12 h. The result Alg/RS films were removed from the glass plates and subjected to further characterization.

##### Thickness

The thickness of the coating films was measured using the digital caliper, and the average of three replicates was determined.

##### Optical properties

The optical properties of the Alg/RS films were measured as described elsewhere^41,42^. The transparency and opacity were measured using the V-730 UV-Visible Spectrophotometer, JASCO, Japan within the wavelength range from 200 to 800 nm.

##### Water susceptibility

The moisture content (MC), water solubility (WS), and the swelling capacity (SC) of the prepared films were supposed to indicate water susceptibility. Films were cut into equal pieces (2 × 2 cm^2^) then weighed for M_0_ and dried at 105 °C to a constant weight (M_1_) and the dried films were soaked for 24 h in 50 mL deionized water. The soaked film samples were dried superficially with filter papers and weight (M_2_). The non-soluble parts were dried at 105 °C to another constant weight (M_3_). Therefore, the MC, SC and WS were calculated according to Costa et al.^43^ as follow:

$$\begin{gathered} {\text{Moisture}}\;{\text{content}}\;\left( \% \right)=\left[ {\left( {{{\text{M}}_0} - {{\text{M}}_{\text{1}}}} \right){\text{ }}/{\text{ }}{{\text{M}}_0}} \right] \times {\text{1}}00 \hfill \\ {\text{Water}}\;{\text{solubility}}\;\left( \% \right)=\left[ {\left( {{{\text{M}}_{\text{1}}}/{{\text{M}}_{\text{3}}}} \right){\text{ }}/{\text{ }}{{\text{M}}_{\text{1}}}} \right] \times {\text{1}}00 \hfill \\ {\text{Swelling}}\;{\text{capacity}}\;\left( \% \right)=\left[ {\left( {{{\text{M}}_{\text{2}}} - {{\text{M}}_{\text{1}}}} \right){\text{ }}/{\text{ }}{{\text{M}}_{\text{2}}}} \right] \times {\text{1}}00 \hfill \\ \end{gathered}$$

##### Film microstructure

The surface and cross-section examination of the prepared Alg films in addition to that prepared by the addition of RS at different concentrations (1, 2.5, and 5%) were carried out using SEM. The gold-coated samples were scanned at 20 kV accelerating voltage and 2000 magnification power under a high-resolution field emission SEM (Quanta 250, HRFEG, Czech).

### Food application of the prepared coating solutions for lemon slice samples

#### Dipping treatment

Prior to coating treatment, the lemon sample was rinsed with distilled water for 1 min, cut into slices with the same dimensions and dipped in the Alg/RS solution for 20 s^44^. The coated slices were left to dry at room temperature for 1 h to remove excess coating solution from sample surface. After complete drying, the coated slices were stored at 4 °C in closed containers. The physicochemical and antioxidant activity of the coated samples were estimated over a storage period of 20 days using the un-coated sample as the control group.

#### Physical properties of the coated lemon samples

The pH, weight loss, and the total soluble solids (TSS) of coated lemon slice samples were periodically estimated. TSS and pH determination were carried out according to AOAC^45^. The weight loss was monitored at zero time and after different storage periods and estimated as following:

$${\text{Weight}}\;{\text{loss}}\;\left( \% \right) = \left[ {\left( {{\text{initial}}\;{\text{weight}} - {\text{final}}\;{\text{weight}}} \right)/{\text{initial}}\;{\text{weight}}} \right] \times {\text{1}}00$$

#### Antioxidant properties of the coated lemon samples

##### Extraction process

All samples were extracted by methanol (70%, 1:10 ratio) for 24 h and the methanolic extracts were centrifuged at 4000 rpm for 15 min. The resulting supernatant was collected and kept at −20 °C until further analysis.

##### Total phenolic content

The total phenolic content (TPC) in the coated lemon extract at different Alg/RS concentrations was assayed by Folin–Ciocâlteu method^33^ using gallic acid standard curve shown in supplementary Fig. 2.

##### Total flavonoid content

The total flavonoid content (TFC) of the coated lemon extract was determined by the aluminum chloride method according to Zhishen et al.^46^. , using rutin standard curve shown in supplementary Fig. 3.

##### Antiradical activity

The antiradical activity of the extract of the coated samples was estimated by DPPH free radical scavenging method according to Dudonne et al.^47^.

###  Statistical analysis

The results were reported as the mean value ± standard deviation for three replicates. ANOVA and the significant differences between the mean values was determined using Duncan test. The level of significance used was *p* < 0.05. Statistical analyses were conducted by using SPSS 20.0 (SPSS for Windows, 2010, SPSS Inc., Chicago, USA).

## Results and discussion

###  Hemicellulose extraction

**Fig. 1:**
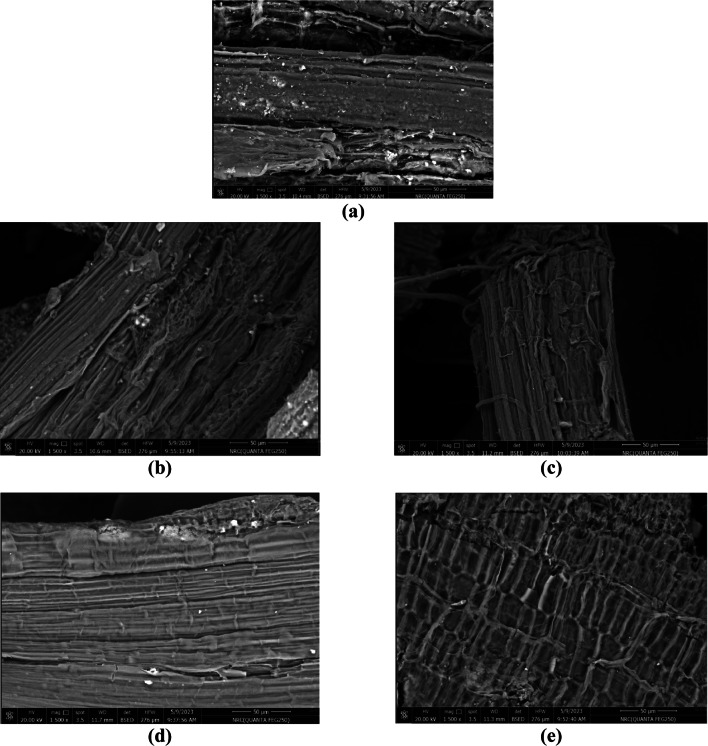
SEM images of (**a**) raw, (**b**) alkaline and (**c**) thermal alkaline treatment of rice straw in addition to microwave treated (**d**) before and (**e**) after alkaline extraction.

Hemicellulose is a natural biopolymer explored in the production of various substances with promising application in food, medical and energy sectors^48^. Herein, extraction of rice straw hemicellulose was carried out using 4% NaOH. The estimated recovery percentage was 13.2% which increased by 19.7% upon thermal treatment. In general, the structure of lignocellulosic biomasses is characterized by high recalcitrant and thermal treatment is one of the most efficient solutions for reducing this recalcitrance and facilitating its utilization^49^. In the current study, SEM images of the crude sample showed the packed structure of plants cell wall that was altered by alkaline treatment with the appearance of the cellulosic bundles (Fig. 1a–c). Upon thermal treatment of the extracting solution, more separation of the cellulosic bundles was observed explaining the increase in the hemicellulose recovery percentage. The obtained recovery percentage was quite similar to that reported by Phan et al.^50^. , under similar extraction conditions. Microwave processing had been estimated as an alternative method for the conventional heating that satisfies many requirements of green chemistry^51^. Therefore, in the current investigation, the extraction of microwave pre-treated rice straw was examined with no significant variation in the recovery percentage. SEM images (Fig. 1d-e) estimated the appearance of attached cellulosic bundles.

**Fig. 2:**
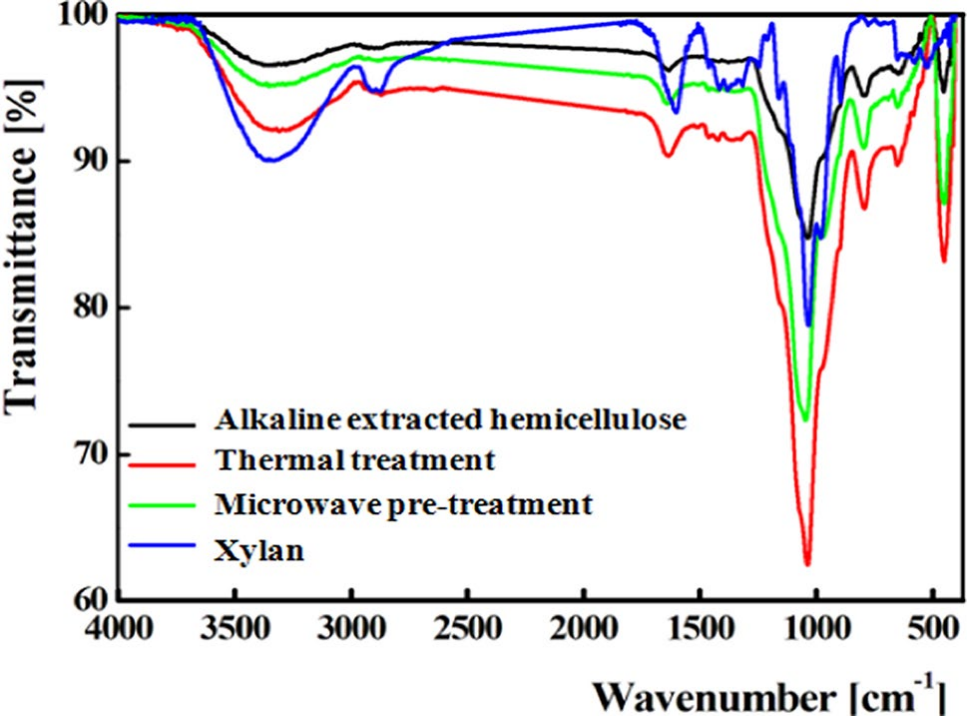
FTIR profile for commercial xylan and the extracted hemicellulose fractions.

FTIR analysis shown in Fig. 2 indicated that the extracted hemicellulose under all the examined conditions possessed the characteristic peaks of commercial xylan in which the identified functional groups were illustrated in Table 1.

**Table 1:** FTIR analysis results.

Wavenumber (cm^−1^)	Functional group
3300	O-H stretching
2900	C-H stretching
1400	C-H, C-OH or C–O bending
1030	C–OH, C–O or C–C stretching
900	beta glycosidic linkage
620	C–C–H stretching
500	C–O–C bending

### Enzymatic hydrolysis of the extracted hemicellulose fraction

The enzymatic activity of the precipitated xylanase fraction was assayed and recorded 407.96 Umg^−1^ protein. *Aspergillus terreus* fungal strain under the described conditions was reported as potent xylanase producer^28^. The production of xylanases was very expressive based on their wide industrial applications^52–55^. The optimum activity conditions for the applied xylanase were estimated at pH 5 and 55 ºC by applying xylan as a substrate (2%, w/v) with a half-life time of 17.3 min at its optimum temperature^52^. Therefore, the enzymatic hydrolysis of the extracted hemicellulose fraction was conducted using 2% hemicellulose in acetate buffer (50 mM, pH 5) at 40 ºC. The estimated hydrolysis yield was 53.80 ± 2.11% which was 1.4-fold higher than the corresponding for the commercial xylan. These results agreed with that previously reported by Hassan et al.^28^.

**Fig. 3:**
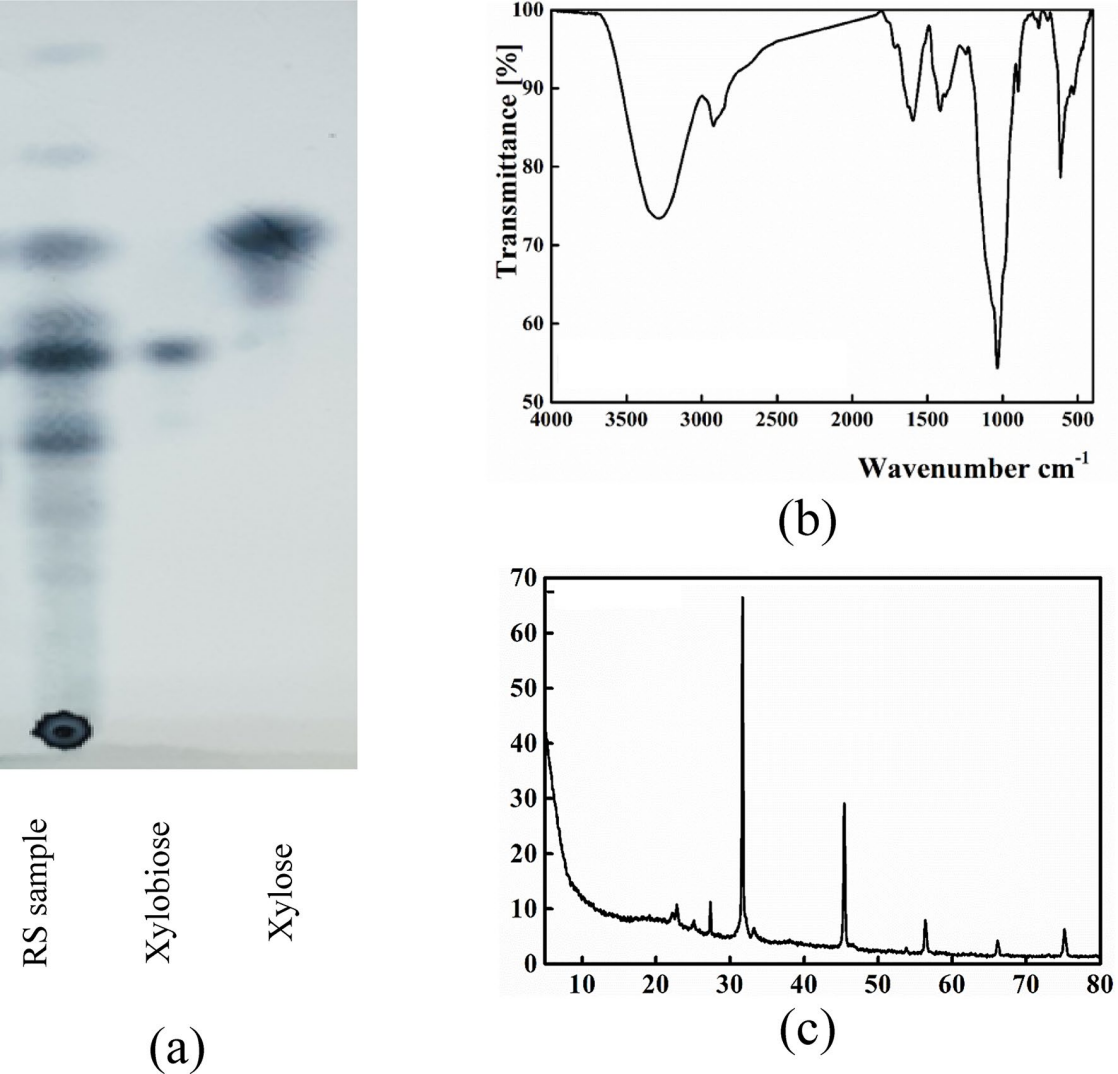
Characterization of RS sample in which TLC (**a**), FTIR (**b**) and XRD (**c**) results wererepresented.

Water solubility of the dried hydrolysate indicated complete solubility of the hydrolysate in comparison to 10% only for the parent compound. Moreover, the total reducing sugars in the dried hydrolysate sample recorded 483.4 ± 9.8 mg xylose/g in absence of a detectable reducing sugar in the parent compound examined at the same concentration. The improvement of the reducing sugar content demonstrated the enzymatic hydrolysis of the glycosidic bonds within the parent compound which led to the release of shorter chains of xylose units which in turn increased the reducing sugars content. TLC analysis showed the release of mono- and oligo-saccharides (Fig. 3a). The produced sugars were assayed by HPLC (supplementary Fig. 4) that indicated, xylobiose (240.68 mg/g) was the main product with the detection of xylose (146.73 mg/g), arabinose (54.95 mg/g) and glucose (36.43 mg/g) with the presence of a minor amount of xylotriose (17.79 mg/g). This result was in agreement with the xylooligosaccharide mixture produced by enzymatic hydrolysis of rice husk alkali-solubilized hemicellulose^56^.

The presence of solubilized lignin phenolic compounds in the extracted hemicellulose fraction was estimated by Amendola et al.^57^. Xiao et al.^58^ indicated that the adding of phenolic compounds improved the antioxidant activity of arabinoxylan in a dose dependent manner. Herein, the phenolic content of the extracted hemicellulose fraction after hydrolysis was found to be 7.4 ± 0.1 mg gallic acid equivalent/g. By HPLC analysis, coumaric (383.33 µg/g) and ferulic (298.77 µg/g) acids were the main identified phenolic compounds (Table 2, supplementary Fig. 5). The recorded result granted with the previously stated results by Menzel et al.^59^ for the extracted phenolic compounds from rice straw.

As shown in Fig. 3b, the produced hydrolysate possessed a similar FTIR pattern with that of the parent compound with the same characteristic peaks reported for xylooligosaccharides^53^. In addition, the XRD pattern (Fig. 3c) of the produced hydrolysate possessed two sharp diffraction peaks at 2θ values 31.7° and 45.4° attributed to the crystallization of xylooligosaccharides and lignin fraction as reported by Sobri et al.^60^.

**Table 2:** Identified polyphenolic compounds in the prepared hydrolysate by HPLC analysis.

Phenolic compound	Concentration (µg/g)
Catechin	38.10
Chlorogenic acid	99.46
Cinnamic acid	6.59
Coumaric acid	383.33
Daidzein	60.30
Ferulic acid	298.77
Gallic acid	48.02
Methyl gallate	10.80
Rutin	41.01
Syringic acid	11.54
Vanillin	20.17

### In vitro cytotoxicity of the dried hydrolysate

The cytotoxicity of the produced hydrolysate examined on normal cells estimated 23.8% viability change at a concentration of 100 ppm, indicating the absence of toxicity of the hydrolysate. From the above results, xylooligosaccharides was estimated as the main components of the resulted hydrolysate. In a close manner, a recent study reported that The European Food Safety Authority (EFSA) has accepted xylooligosaccharides as a novel food, and the FDA has generally recognized them as safe for use in foods, with no toxic effects at a single dose of 5 g/Kg body weight in Wistar rats^61^.

### Antioxidant activity of the dried hydrolysate

**Fig. 4:**
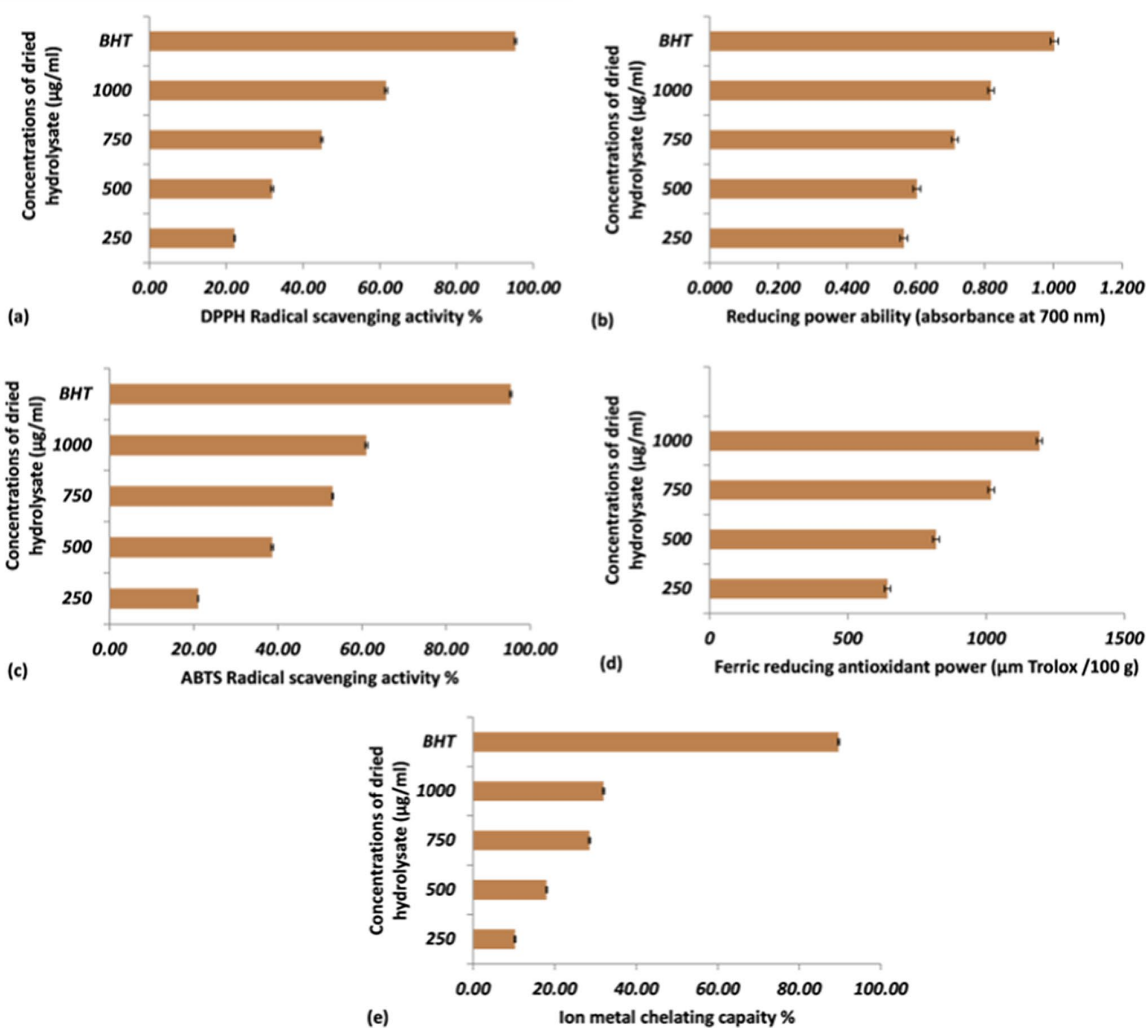
Antioxidant activity of different hydrolysate concentrations applying different antioxidant assay methods: (**a**) scavenging ability on DPPH radical, (**b**) reducing power ability, (**c**) scavenging ability on ABTS, (**d**) ferric reducing antioxidant power, (**e**) ion metal chelating activity.

In food production, excess oxidation is a crucial problem as it causes spoilage and rancidity of food products^62^. Moreover, the negative impact of oxygen-free radicals on aging, cancer pathogenesis, cardiovascular diseases and neurological disorders had been explored in the last few decades, which had raised a lot of attention in the antioxidant activity of food and diet with a great scope on natural antioxidants^63–65^. For many years, extracts of plant origin rich in antioxidant compounds had been applied to increase the shelf-life of the final food products and provide human health advantages^66^. In this concern, utilization of various byproducts in order to produce compounds with antioxidant activity applied in food production had attracted the research focus^67^. The antioxidant activity of the produced hydrolysate was measured at different concentrations (250–1000 µg/ml) using various methods (DPPH, ABTS, reducing power, FRAP and Ion metal chelating). The results presented in Fig. 4a–e confirmed that the increasing of the concentration of the sample was directly proportional with its antioxidant activity. The concentration of 1000 µg/ml achieved high antioxidant activity of 61.58 ± 0.36% DPPH radical scavenging activity, 0.818 ± 0.009 (absorbance at 700 nm) reducing power ability, 61.01 ± 0.32% ABTS radical scavenging capacity, 1192 ± 10.54 µmol Trolox/100 g ferric reducing antioxidant power, and 31.99 ± 0.20% ion metal chelating. The standard antioxidant BHT achieved 95.35 ± 0.27% DPPH radical scavenging activity, 1.003 ± 0.012 (absorbance at 700 nm) reducing power ability, 95.39 ± 0.242% ABTS radical scavenging capacity, and 89.67 ± 0.23% ion metal chelating. Meanwhile, the concentrations 500 and 750 µg/ml exhibited a good antioxidant activity by recording 44.85 ± 0.29 and 31.96 ± 0.37% DPPH radical scavenging activity, 0.713 ± 0.010 and 0.603 ± 0.011 reducing power ability, 53.01 ± 0.16 and 38.67 ± 0.28% ABTS radical reducing power, 1018 ± 11.50 and 819 ± 11.93 µmol Trolox/100 g ferric radical antioxidant power, and 28.53 ± 0.24 and 18.03 ± 0.11% ion metal chelating, respectively. The high antioxidant activity of the produced hydrolysate could be explained on the basis of xylooligosaccharide and polyphenolic contents^68,69^.

### Characterization of alginate/rice straw-hemicellulose hydrolysate films

#### Thickness

In general, the thickness of a synthesized film is a crucial issue which affects different mechanical and barrier characteristics of the packaging materials. Herein, the thickness of the investigated films was evaluated as tabulated in Table 3. A steady increase was noticed by increasing the percentage of the added RS. Similar behavior had been estimated by increasing the concentration of the added substance within the matrix of sodium alginate/carboxymethyl cellulose films. This increase could be attributed to the enhanced interaction (hydrophobic interaction and hydrogen bonding) between the added substance and the matrix constituents leading to the construction of a more compact film structure and an increase in the film thickness^70^.

**Table 3:** The thickness and the UV-visible measurements of Alg film blended with different concentrations of RS.

Film samples	Thickness (mm)	% T 660 nm	Abs 600 nm
Alg (0% RS)	0.050 ± 0.001^d^	49.03 ± 0.03^a^	0.34 ± 0.02^d^
Alg/RS 1 (1% RS)	0.052 ± 0.002^c^	41.27 ± 0.02^b^	0.48 ± 0.01^c^
Alg/RS 2.5 (2.5% RS)	0.068 ± 0.003^b^	37.09 ± 0.01^c^	0.49 ± 0.01^b^
Alg/RS 5 (5% RS)	0.074 ± 0.002^a^	30.04 ± 0.06^d^	0.65 ± 0.04^a^

#### Optical properties

**Fig. 5:**
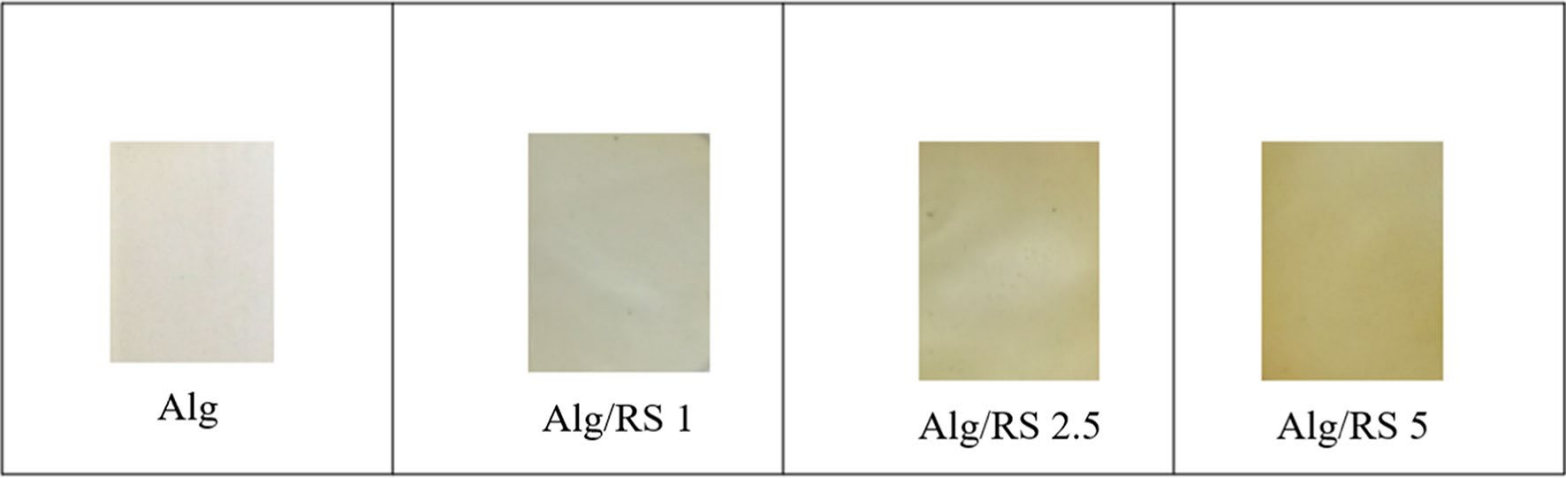
Visual appearance of Alg and Alg/RS films with different RS concentrations of 1, 2.5 and 5%, respectively.

The visual apparition of the prepared films clearly signified that the control Alg film was bright yellow, while the Alg/RS films color changed gradually to bright brownish-yellow with the addition of different RS concentrations of 1, 2.5 and 5% (Fig. 5).

The prepared films optical properties were presented in Table 3 and supplementary Fig. 6. The results indicated a reduction in the light transmittance range that increased by increasing the percentage of RS in the film. A high % T value indicated that the film was more transparent and lighter in which the light could effectively pass through the film. Generally, the packaging films optical properties are essential issues for choosing the suitable packaging materials for the good food product^71^.

#### Water susceptibility

The results of water susceptibility of the prepared films were illustrated in Table (4). The water content ranged from 20.382 to 21.121%. Mustapa et al.^72^. , reported that the eco-friendly films showed moisture content values between 16.48 and 23.96%. The moisture content of an edible film is directly influenced by the total amount of dissolved compounds in it. According to Khwaldia et al.^73^, date palm extract incorporation to alginate film notably increased the water contact angle (*p* < 0.05) because of the extract polar compounds. In the current study, the water content of Alg/RS films significantly (*p* < 0.05) increased with the increase of RS content.

The water solubility of the Alg-control film may be ascribed to the presence of hydroxyl and carboxyl groups within the alginate molecules. The water solubility of the Alg/RS films increased significantly from 76.103 to 98.563% (*p* < 0.05) with the increasing amount of RS in the films. The gradual enhancement in the solubility of Alg/RS films could be owing to the increase in the hydrophilic groups of the polyphenolic content of RS. Sun et al.^74^, reported similar results, indicating an enhancement in the water solubility of the thinned young apple polyphenols-chitosan films as the hydrophilic groups of the polyphenols structure act together with water molecules.

Swelling of the Alg/RS films significantly reduced upon increasing the content of the RS hydrolysate (*p* < 0.05). There was a possibility that RS polyphenols’ interaction with alginate’s carboxyl groups would decrease its water accessibility. Thus, the ability of the Alg/RS films to swell might have been restricted by increasing RS concentration. The same swelling trend was described by Aydin & Zorlu^75^, on composite films composed of alginate and different concentrations of roselle extract (1, 3, and 5% w/v) in which the swelling capacity was found to be 4591.42, 1320.44 and 484.19%, respectively.

**Table 4:** Water content, solubility and swelling capacity of Alg and Alg/RS films.

Film samples	Water content (%)	Water solubility (%)	Swelling capacity (%)
Alg (0% RS)	21.374 ± 0.118 ^a^	84.853 ± 1.002^c^	482.581 ± 1.597^a^
Alg/RS 1 (1% RS)	20.382 ± 0.058 ^c^	76.103 ± 0.503^d^	425.561 ± 5.741^b^
Alg/RS 2.5 (2.5% RS)	20.844 ± 0.151 ^b^	89.996 ± 0.754^b^	158.896 ± 2.788^c^
Alg/RS 5 (5% RS)	21.121 ± 0.166 ^a^	98.563 ± 0.500^a^	63.557 ± 1.693^d^

#### FTIR spectroscopy

**Fig. 6:**
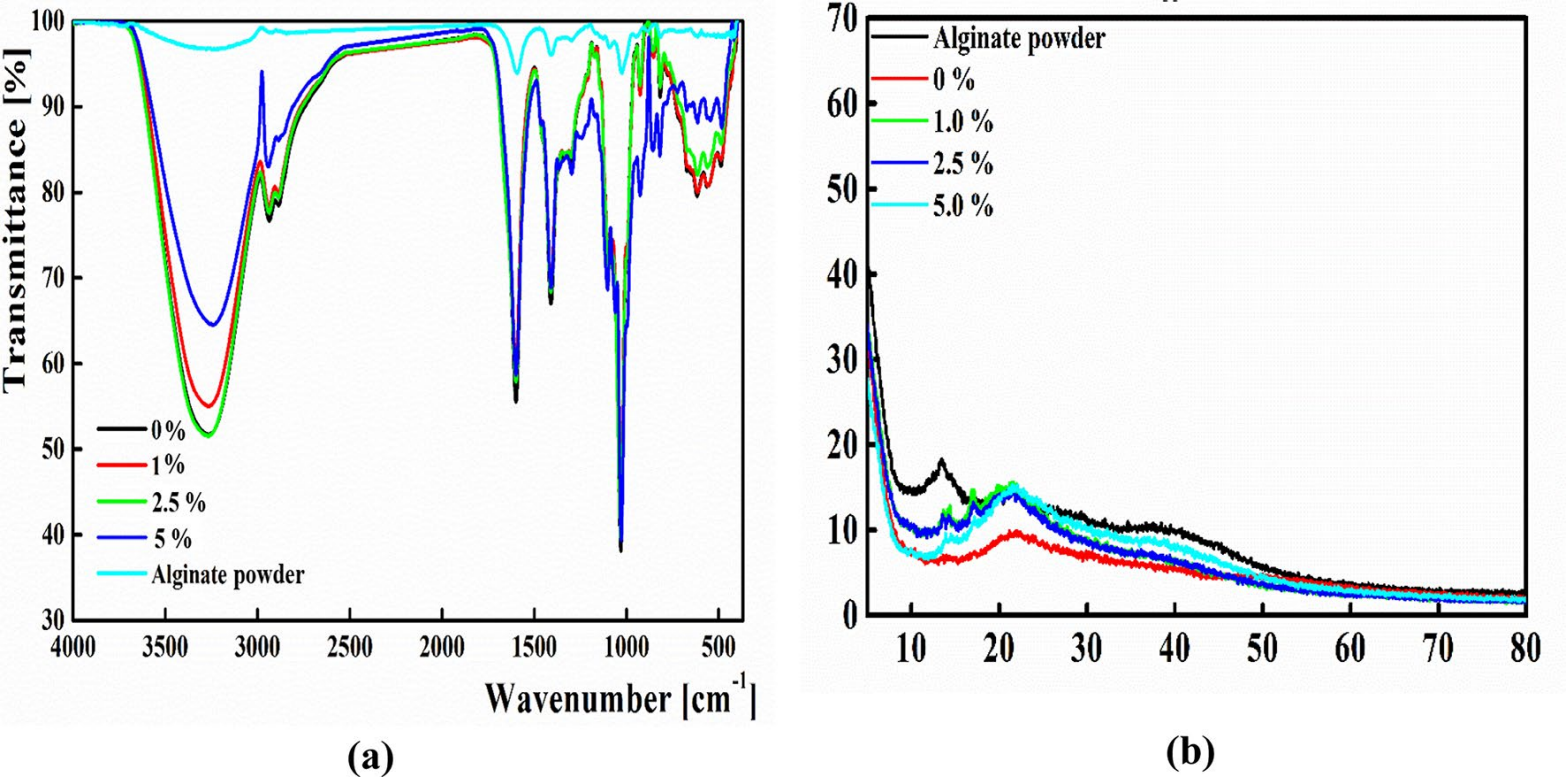
(**a**) FTIR spectra, (**b**) and XRD pattern of alginate powder and Alg films prepared using different concentrations of RS (0, 1, 2.5, and 5%).

The FTIR spectra of sodium alginate compared to those integrated with RS were illustrated in Fig. 6a. Sodium alginate powder exhibited a characteristic peak at 1591.08 cm^−1^ corresponded to the asymmetric stretching modes of carboxylate anion with a broad peak at 3232.31 cm^−1^ corresponded to the stretching vibration of the hydroxyl groups^76^. Although the peak at 1413.65 cm^−1^ corresponded to the symmetric stretching vibration of the COO groups, the peak at 1027.93 cm^−1^ related to the symmetric elongation of C-O groups, and the peak at 819.64 cm^−1^ related to the symmetrical C-O-C stretching^77^.

Moreover, the Alg film possessed five pronounced peaks at 3262, 2935, 1599, 1409 and 1029 cm^−1^. The broad peak at 3262 resulted from the stretching vibration of -OH groups. In addition, the peak estimated at 2935 cm^−1^ resulted from the stretching vibration of C-H bonds. The peaks appeared at 1599 cm^−1^ and 1409 cm^−1^ resulted from the symmetric and asymmetric stretching vibration of the COO groups, respectively. The peak at 1029 cm^−1^ corresponded to the stretching of the C–O–C glycosidic bond^78^. The Alg/RS films prepared by blending different RS concentrations (1, 2.5, and 5%) with Alg possessed the same characteristic peaks of Alg film, estimating no chemical interaction between the film constituents. Martins et al.^79^ described analogous results for the addition of xylooligosaccharides to the alginate-gelatin blend.

#### XRD analysis

The XRD pattern of sodium alginate and the Alg/RS films were presented in Fig. 6b. Sodium alginate powder possessed two distinct peaks at 2θ values of 13.5° and 20.7° corresponded to the refraction of its amorphous (110) plane from the glucuronate unit and (200) plane from the polymannuronate unit^80^. By comparing the XRD pattern of the prepared Alg/RS films with that of the Alg film, no alteration in the crystal structure of the edible film was observed, but the peak movement degree became higher as the concentration of the added RS increased. This result demonstrated that the RS had good compatibility with alginate, and the addition of RS didn’t impact the original structure of the film. Identical manner had been estimated previously by Abdin et al.^81^ and Akhtar et al.^70^ for the addition of *Thymus vulgaris* leaf extract and phycocyanin, respectively within sodium alginate/carboxymethyl cellulose films.

#### Scanning electron microscopy

**Fig. 7:**
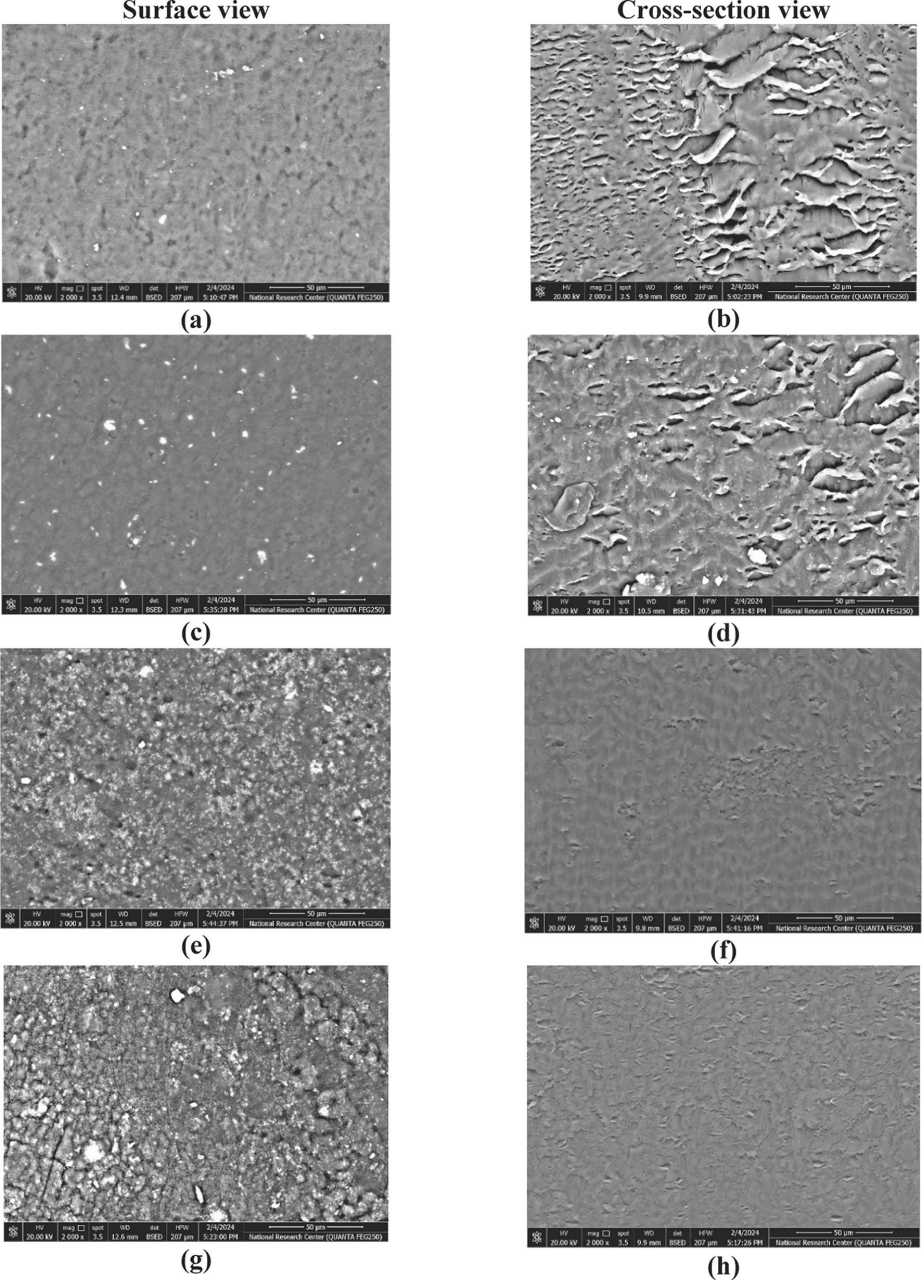
Surface and cross-section SEM results of Alg film (**a**, **b**) in addition to that with the adding of different concentrations of RS 1% (**c**, **d**), 2.5% (**e**, **f**) and 5% (**g**, **h**).

The surface and cross-section views of the prepared Alg and Alg/RS films were presented in Fig. 7. The results point to significant variances between the Alg film with and without the addition of RS. The Alg film possessed a uniform surface with wide pores within the constructive matrix of the film estimated in the cross-section view. By the addition of the RS, the film surface converted to be rougher and irregular with the visibility of granular vesicles by increasing the concentration of the added RS to 5%. Moreover, the cross-section view estimated the gradual disappearance of the pores with the formation of a condensed matrix by increasing the concentration of RS. Similar behavior was estimated for alginate-based films blended with laurel leaf extracts and olive leaf^78^.

### Food application of the prepared coating solutions for lemon slice samples

#### Quality evaluation

**Fig. 8:**
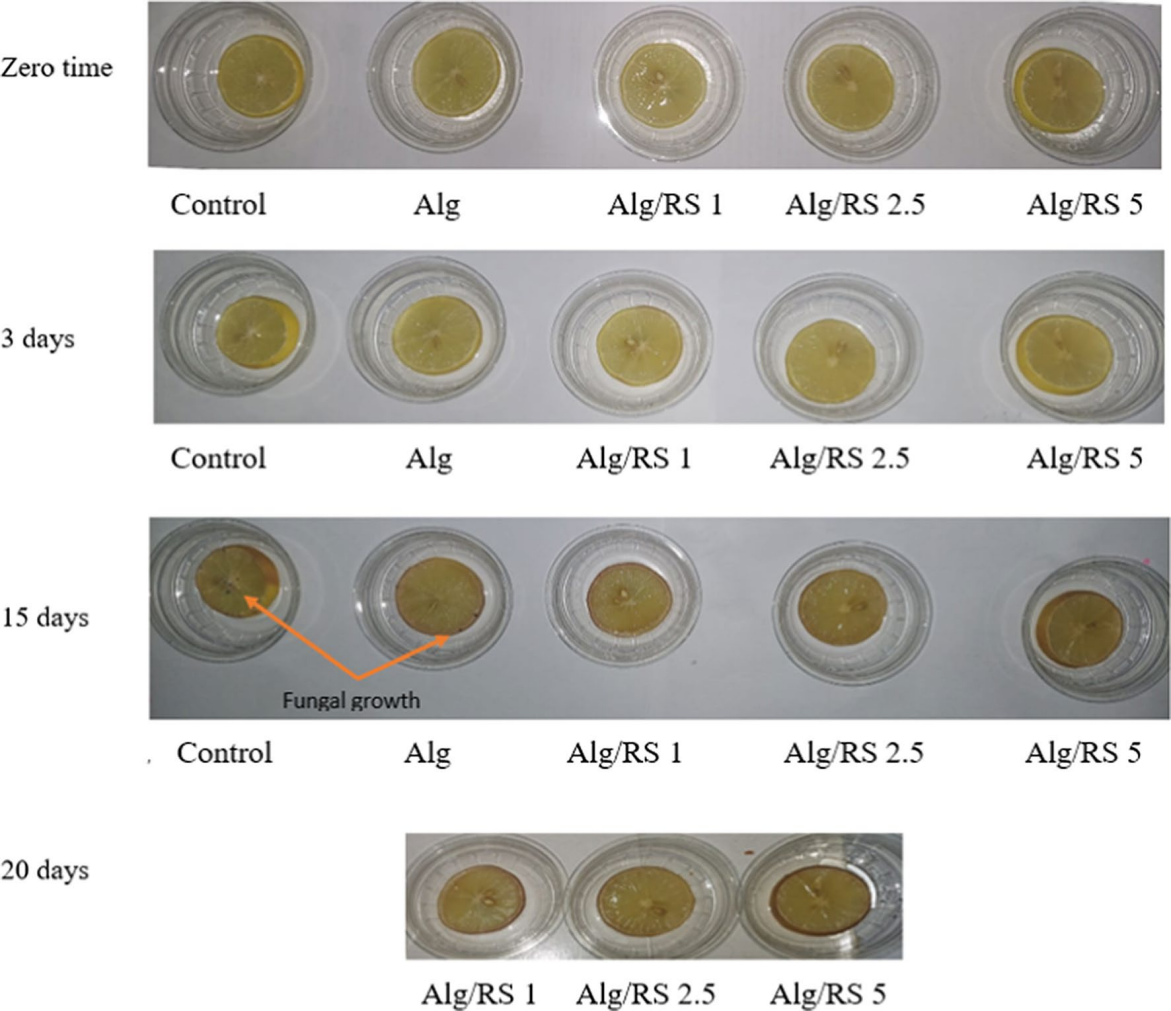
Visual appearance of lemon slice non coated (control), coated with sodium alginate dipping solution (Alg) and Alg/RS at various concentrations (1, 2.5 and 5%) as (T1, T2 and T3).

Initially, the appearance of the lemon samples was evaluated and it was obviously estimated that the uncoated samples began to dry out and deteriorate by the 7th day of storage compared to the coated ones. In addition, fungal growth was visually observed by the 15th day of storage in the untreated samples as well as the samples coated with Alg film, in contrast to those coated with Alg/RS films of different RS concentrations (1, 2.5, and 5%) (Fig. 8). After that, the lemon samples were evaluated for weight loss, changes in pH, and TSS change.

##### Weight loss

**Fig. 9:**
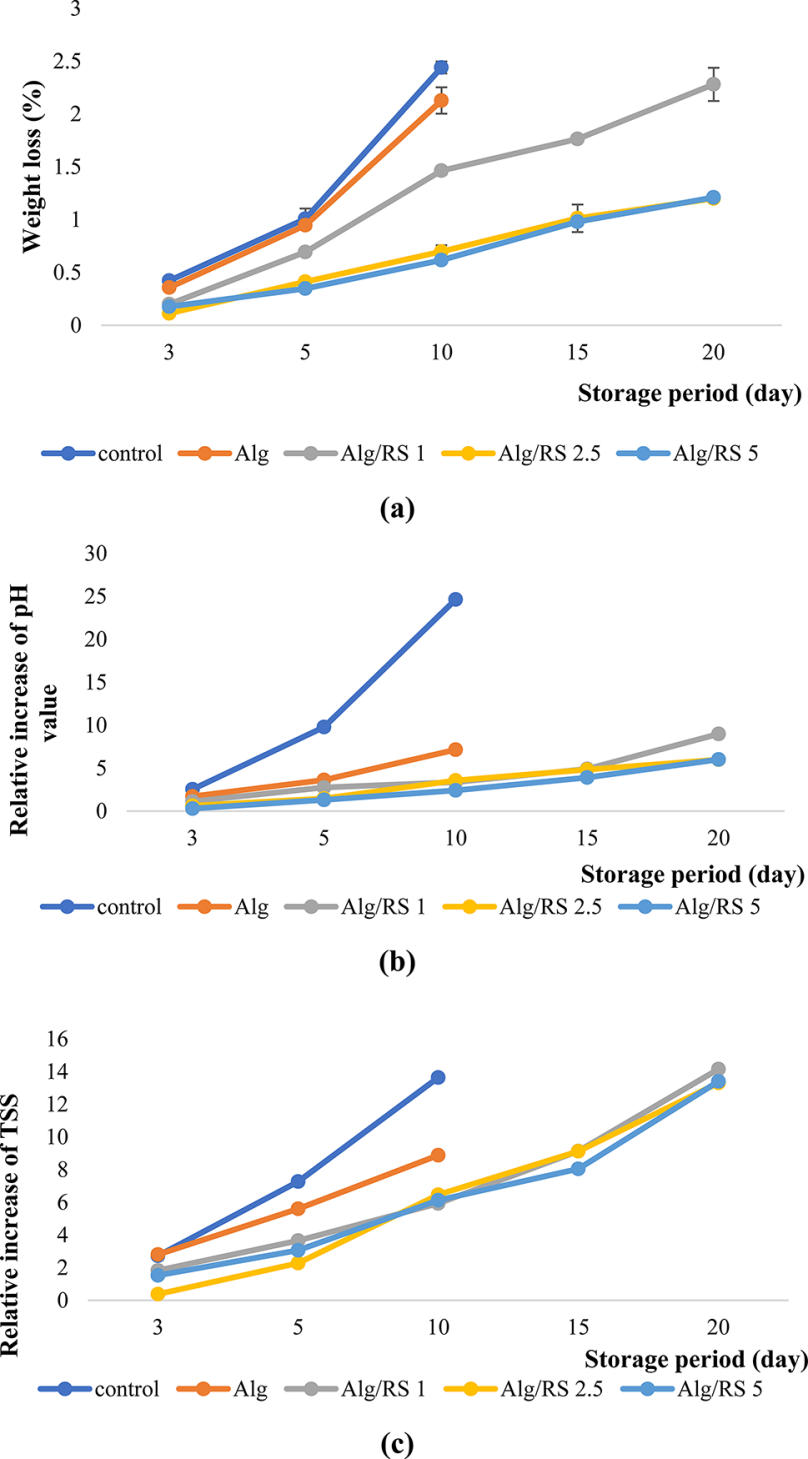
The effect of Alg/RS coating with different RS concentrations (1, 2.5, 5%) on (**a**) weight loss, (**b**) pH and (**c**) TSS of lemon samples during 20 days of storage at 4 ± 1 °C.

In general, weight loss of the stored samples is a crucial variable in determining the quality of vegetables and fruits which increases along the storage period^82^. Applying either dipping or spraying method, fresh fruits and vegetables are typically covered with a thin layer on their surface which functions as a semipermeable membrane and prevents moisture loss. In the following study, the weight loss of the coated lemon slice samples was evaluated which increased over time for all treatments but a significant reduction in weight loss was observed for samples coated by Alg/RS 2.5, and 5% (Fig. 9a). Guimarães et al.^83^ explained that, even under perfect storage circumstances, the combined effects of transpiration and respiration led to an increase in mass loss for all fresh-cut carrot treatments over the course of the 28-day storage period.

##### Change of pH

The pH relative change of lemon samples influenced by the Alg/RS film during storage stage at 4 ± 1 °C was monitored. The pH of the coated samples increased rapidly throughout the storage period in the uncoated samples while, the rate of increase decreased in all coating treatments. The rate of increase in the pH values reached 7.16% in Alg coated samples after 10 days of storage, while the addition of RS in the coating solution slowed down the rate of increase as shown in Fig. 9b and supplementary Table 1. In general, organic acids constitute the majority of the H^+^ ions. Nasrin et al.^84^ reported that increasing the pH values of coated lemon fruits through the storage time was controlled by the consumption of organic acids during respiration. Chau et al.^85^ and Goswami et al.^82^ estimated that the coating of citrus fruits slowed the shift in the pH, indicating its preservative effect for essential and beneficial organic acid constituents.

##### Total soluble solids content

The TSS content represents the amount of sugars, acids, minerals and vitamins in addition to amino acid constituents of vegetables and fruits^86^. The fruit’s TSS concentration will be impacted by the enhancement of physiological processes during storage, such as its respiratory metabolism. Figure (9c) elucidated the impact of Alg/RS edible coating on the TSS of lemon samples during storage period at 4 ± 1 °C. The TSS content of lemon samples in all treatments showed a trend of increase that was rapid in un-coated samples, followed by samples coated with alginate only, where the rate of increase after 10 days of storage reached 13.64% and 8.879%, respectively. The slower increase in the TSS of the coated samples (T1, T2 and T3) could be attributed to the formation of a physical barrier slowing the respiration processes that led to the solubilization of the complex constituents as a result of their depolymerization and formation of simple constituents^70^. The same trend was observed by Kefayatullah & Wahab^87^, and Chau et al.^85^, indicating an increase in the TSS values of the coated fruits during the storge period. Chen et al.^88^ reported a trend of increasing for the TSS content of coated strawberries which decreased at the end of the storage time.

After 15th day of storage, lemons in groups T1, T2 and T3 still had a controlled TSS content while, at the end of the storage period there were no significant changes in the rate of increase of TSS in the T2 and T3 samples (supplementary Table 1). From the above results, it could be concluded that the applied coating via the addition of different concentrations of RS enhanced the reduction of the nutrients loss during the storage period without a noticeable difference between the use of 2.5 and 5%. Therefore, the use of 2.5% could be considered the best applied concentration.

Lemon slice samples covered with alginate only, despite their stability during storage in comparison to the un-coated samples, the appearance of fungal growths after 10 days of storage limited the possibility of using sodium alginate alone for covering. Moreover, considering the previous results of weight loss, pH, and TSS for all treatments, it could be concluded that the increment of RS in the coating solution improved the shelf-life of the coated lemon samples stored in the refrigerator within 20 days. After this period, although there was no deterioration or decrease in the values of both the pH and TSS of the coated samples, they began to appear in an undesirable form with a brown color appearing.

#### Antioxidant properties

##### Total phenolic content

**Fig. 10:**
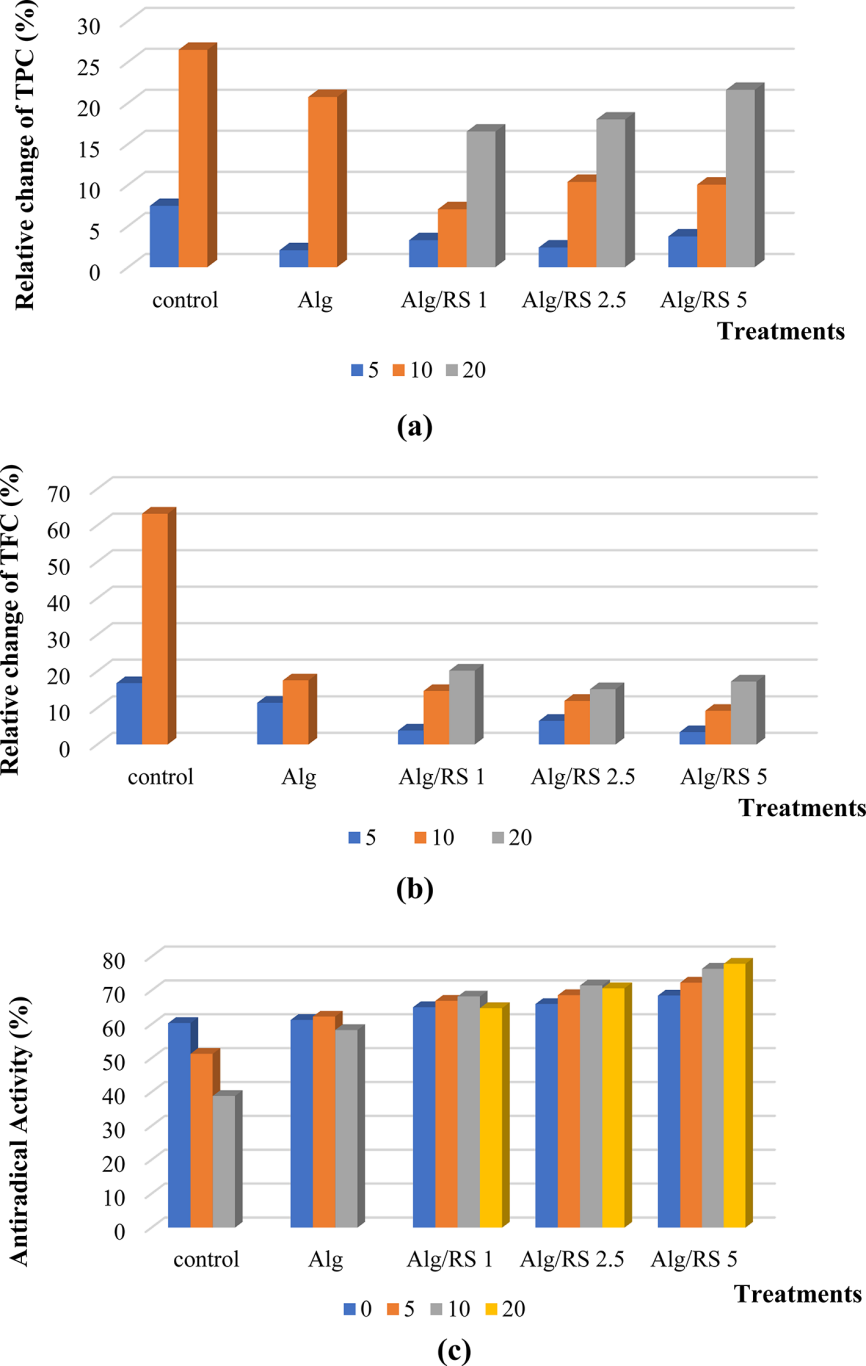
Relative change of (**a**) TPC and (**b**) TFC of lemon slices; and (**c**) the antiradical activity of lemon samples during 5, 10, 20 days of storage at 4 ± 1 °C.

In general, TPC is strongly correlated with the antioxidant activity which is mediated by the redox and free-radical scavenging activity displayed by its constituents. The change of TPC of coated lemon samples throughout the storage period was presented in Fig. (10a). The TPC of the samples gradually decreased throughout the storage period and the highest rate of reduction was observed in the un-coated samples that significantly lowered by coating. Moreover, the mean values of TPC decreased from 4.27, 4.17 and 4.36 to 3.56, 3.33 and 3.42 (mg GE/g) with a relative decrease of 16.50, 17.97 and 21.58% for sample coated with Alg/RS 1, 2.5, and 5%, respectively, compared with a relative decrease of 26.46 and 20.71% for the un-coated and Alg coated samples, respectively (Supplementary Table 1). Similar studies reported that the coating could preserve the phenolic contents by controlling the oxygen migration and the preservative effect of the coating on the TPC of fruits^89^.

##### Total flavonoid content

The results estimated that the minimum diminish in the TFC was recorded for the sample coated with Alg/RS 2.5% and the maximum decrease for TFC was observed in the un-coated lemon sample (Fig. 10b). Statistical analysis pointed that the coating treatments had TFC significant impact (supplementary Table 1).

##### Antiradical activity

The antiradical activity (AA) of both uncoated and coated samples was monitored as represented in Fig. 10c, and supplementary Table 1. When the coated samples were compared with the uncoated samples during the storage period, an increase in the antiradical activity percentage was estimated with a significant decrease in the activity percentage in the uncoated samples. Moreover, a concentration dependent increase in the activity was estimated for RS containing coated samples. These results were in harmony with the previous reports estimated for the coating of citrus fruits with edible coatings functionalized with antioxidant-rich compounds^89^.

## Conclusion

In the present study, efficient extraction of rice straw-hemicellulose was carried out under thermal alkaline conditions followed by enzymatic hydrolysis, producing a water-soluble hydrolysate possessed a high content of xylooligosaccharides and phenolic compounds with potent antioxidant activity. The prepared hydrolysate was incorporated with alginate for the preparation of food preservative coatings solutions and edible films. Finally, the prepared mixture was applied to preserve lemon slices. Considering the results presented previously for quality parameters and antioxidant properties of lemon slices coated with Alg/RS dipping solutions, it turned out to be a successful strategy to blend rice straw-hemicellulose hydrolysate with sodium alginate to manufacture an edible food film that helped in prolonging fresh foods shelf-life. In addition, a sodium alginate dipping solution with 2.5% hemicellulose hydrolysate was considered the most appropriate for practical food applications. This research revealed the opening of a new field for the possible utilization of agro-industrial byproducts in many food applications, which will likewise be helpful in waste management and environmental safety.

## Electronic Supplementary Material

Below is the link to the electronic supplementary material.

Supplementary Material 1

## Data Availability

All data generated or analysed during this study are included in this published article and its supplementary information files.
