# Supplementary material for: Preservation of freshly-cut lemon slices using alginate-based coating functionalized with antioxidant enzymatically hydrolyzed rice straw-hemicellulose
Source: Sci Rep. 2024 Nov 8;14:27176. doi: 10.1038/s41598-024-77670-6 (PMC11543928; doi:10.1038/s41598-024-77670-6)
Supplement: Supplementary file 1 — Supplementary Material 1 [file 41598_2024_77670_MOESM1_ESM.docx]

|  |
| --- |
| Supplementary figure (1): Standard curve of xylose for the estimation of the enzyme activity. |

|  |
| --- |
| Supplementary figure (2): Standard curve of gallic acid for the estimation of the total phenolic content. |

|  |
| --- |
| Supplementary figure (3): Standard curve of rutin for the estimation of the total flavonoid content. |

| **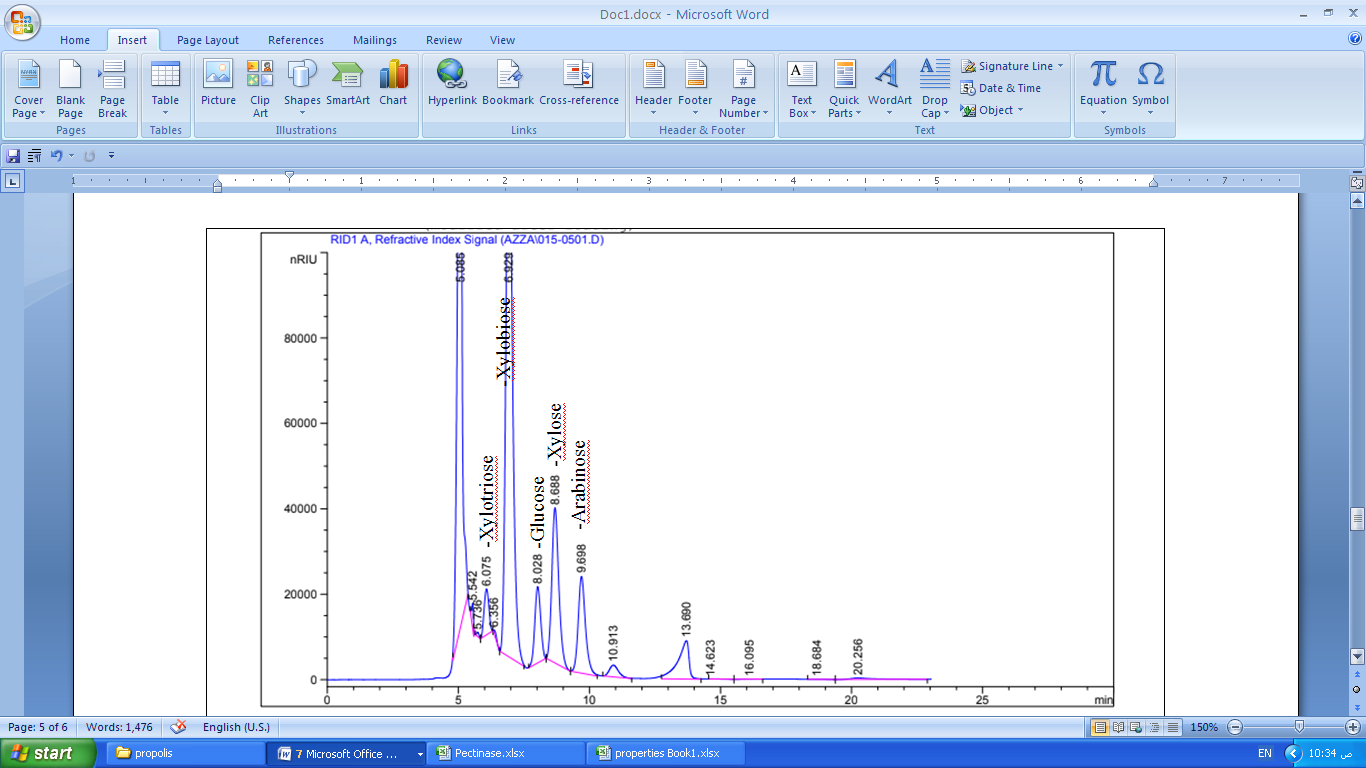** |
| --- |
| Supplementary figure (4): HPLC chromatogram for sugars present in RS hydrolysate |
| 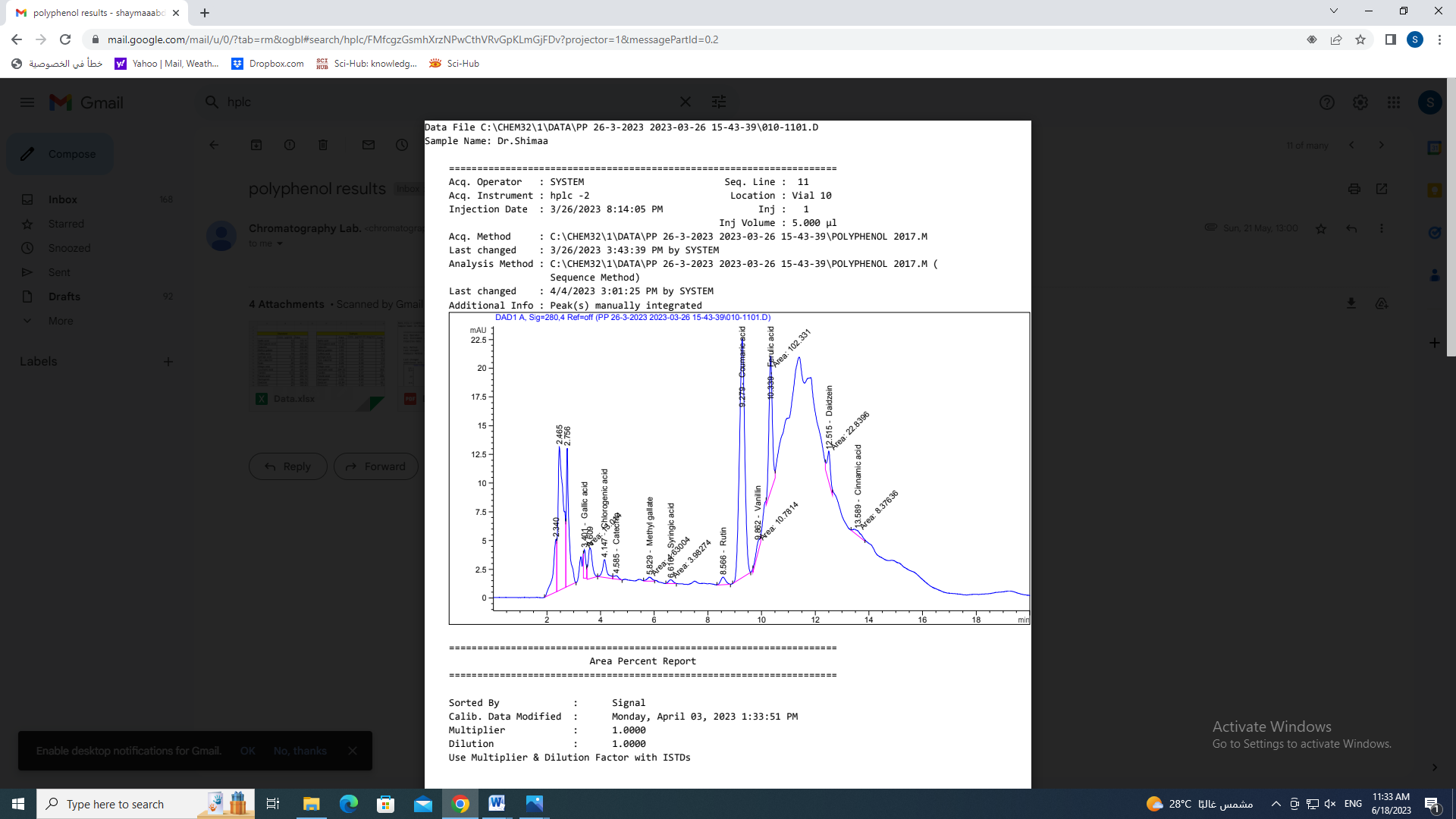 |
| Supplementary figure (5): HPLC chromatogram for polyphenolic content present in RS hydrolysate |

**Supplementary table (1):** **Change in pH, TSS (Brix°), TPC, TFC and antiradical activity of uncoated and coated lemon during 20 days of storage (4 ± 1 °C)**

| Storage period  (days) | Treatments | | | | |
| --- | --- | --- | --- | --- | --- |
|  | **Un-coated** | **Alg** | **T1** | **T2** | **T3** |
| pH | | | | | |
| 0 | 3.27^d^ | 3.31^d^ | 3.27^f^ | 3.17^f^ | 3.32^e^ |
| 3 | 3.36^c^ | 3.36^c^ | 3.30^e^ | 3.19^e^ | 3.33^e^ |
| 5 | 3.59^b^ | 3.43^b^ | 3.36^d^ | 3.22^d^ | 3.37^d^ |
| 10 | 4.08^a^ | 3.54^a^ | 3.38^c^ | 3.29^c^ | 3.40^c^ |
| 15 | ND | ND | 3.43^b^ | 3.33^b^ | 3.45^b^ |
| 20 | ND | ND | 3.56^a^ | 3.36^a^ | 3.52^a^ |
| TSS (%) | | | | | |
| 0 | 7.7^c^ | 7.1^d^ | 7.3^d^ | 8.8^e^ | 8.7^e^ |
| 3 | 7.9^d^ | 7.3^c^ | 7.4^d^ | 8.8^e^ | 8.8^e^ |
| 5 | 8.3^b^ | 7.5^b^ | 7.6^c^ | 9.0^d^ | 9.0^d^ |
| 10 | 8.7^a^ | 7.8^a^ | 7.7^c^ | 9.3^c^ | 9.2^c^ |
| 15 | ND | ND | 8.0^b^ | 9.6^b^ | 9.4^b^ |
| 20 | ND | ND | 8.3^a^ | 9.9^a^ | 9.9^a^ |
| TPC (mg GE/g) | | | | | |
| 0 | 4.04^a^ | 3.94^a^ | 4.27^a^ | 4.17^a^ | 4.36^a^ |
| 5 | 3.74^b^ | 3.86^b^ | 4.13^b^ | 4.07^b^ | 4.20^b^ |
| 10 | 2.97^c^ | 3.13^c^ | 3.97^c^ | 3.74^c^ | 3.93^c^ |
| 20 | ND | ND | 3.56^d^ | 3.33^d^ | 3.42^d^ |
| TFC (mg QE/g) | | | | | |
| 0 | 0.64^a^ | 0.82^a^ | 0.71^a^ | 0.81^a^ | 0.80^a^ |
| 5 | 0.53^b^ | 0.73^b^ | 0.69b | 0.78^b^ | 0.75^b^ |
| 10 | 0.23^c^ | 0.68^c^ | 0.61^c^ | 0.74^c^ | 0.70^c^ |
| 20 | ND | ND | 0.57^d^ | 0.67^d^ | 0.68^d^ |
| Antiradical activity (%) | | | | | |
| 0 | 60.34^a^ | 61.26^b^ | 65.04^c^ | 65.99^d^ | 68.48^d^ |
| 5 | 51.26^b^ | 62.26^a^ | 66.93^b^ | 68.58^c^ | 72.27^c^ |
| 10 | 38.81^c^ | 58.29^c^ | 68.21^a^ | 71.45^a^ | 76.38^b^ |
| 20 | ND | ND | 64.77^d^ | 70.63^b^ | 77.85^a^ |

| **(a)** | |
| --- | --- |
| **(b)** | |
| **(c)** | **(d)** |
| Supplementary figure (6): UV–visible spectra of Alg/RS films; (a) transmittance and (b) absorption spectra in addition to the optical properties of the films; (c) transparency at 660 nm and (d) opacity at 600 nm in which the Alg film was the control and T1, T2 and T3 were Alg/RS films with different RS concentrations of 1, 2.5 and 5 %, respectively. | |
